# Amino acid residues in the tail fiber differentiate the host specificity of *Cronobacter sakazakii* bacteriophage

**DOI:** 10.1128/jvi.00289-25

**Published:** 2025-04-11

**Authors:** Eunshin Cho, Jinshil Kim, Nam-Chul Ha, Sangryeol Ryu

**Affiliations:** 1 Department of Food and Animal Biotechnology, Research Institute of Agriculture and Life Sciences, Seoul National University 26725, Seoul, Republic of Korea; 2 Department of Agricultural Biotechnology, Seoul National University 539783, Seoul, Republic of Korea; 3 Center for Food and Bioconvergence, Seoul National University 26725, Seoul, Republic of Korea; 4 Department of Food Science and Biotechnology, Sejong University 35006, Seoul, Republic of Korea; 5 Carbohydrate Bioproduct Research Center, Sejong University 35006, Seoul, Republic of Korea; Michigan State University, East Lansing, Michigan, USA

**Keywords:** bacteriophage, *Cronobacter sakazakii*, tail fiber, lipopolysaccharide, O serotype

## Abstract

*Cronobacter sakazakii* is a Gram-negative pathogen that causes severe infections such as neonatal meningitis and sepsis. Bacteriophages (phages) rely on tail fibers for host recognition and infection, but the mechanisms of how phages recognize their bacterial hosts are not completely elucidated. In this study, two lytic *C. sakazakii* phages belonging to the *Drexlerviridae* family, CRES7 and CRES9, were isolated from sewage in South Korea. The genomes of both phages are almost the same, with only two nucleotide differences in the gene encoding a putative tail fiber, causing two amino acid differences at amino acid residues 400 and 550 of the tail fiber. The predicted structure of the tail fiber revealed that the two amino acid residues are located on the surface of the tail fiber, suggesting that these two amino acid residues may affect receptor binding. These amino acid differences resulted in differential host ranges, adsorption rates, and burst sizes of CRES7 and CRES9; CRES7, which could infect only the *C. sakazakii* serotype O1 strain, exhibited a higher adsorption rate and larger burst size compared to CRES9, whereas CRES9 could infect both serotypes O1 and O3 strains. These findings provide insights into how the mutations in the tail fiber gene contribute to the fitness of phages within natural environments and help develop phage-based strategies with expanded host range or enhanced specificity for targeted biocontrol of *C. sakazakii*.

**IMPORTANCE:**

Accurate recognition and attachment to the bacterial host, mediated by tail fibers, are crucial for successful phage infection. Understanding the mechanisms underlying host specificity of phages is essential for developing targeted biocontrol applications. This study identified specific amino acid residues responsible for host specificity in the tail fibers of two newly isolated *Cronobacter sakazakii* phages, CRES7 and CRES9. Differences in these residues showed variation in O serotype recognition, leading to differences in host range, adsorption efficiency, and burst size. These findings provide valuable insights into tail fiber-mediated host specificity, facilitating the development of more effective phage-based strategies against *C. sakazakii*.

## INTRODUCTION

*Cronobacter sakazakii* is a Gram-negative opportunistic foodborne pathogen widely distributed in the environment (1). *C. sakazakii* is frequently isolated from various foods, such as powdered infant formula, dried fish, grains, and dairy products (2, 3). This pathogen poses a significant public health concern due to its capacity to induce severe infections, including neonatal meningitis and necrotizing colitis with reported mortality rates as high as 80% (4). It also threatens elderly individuals and immunocompromised patients (5, 6), emphasizing the need for effective control measures against *C. sakazakii*. While antibiotics have been traditionally used as antimicrobial agents, the emergence of antibiotic-resistant bacteria has raised the necessity for alternative antimicrobial strategies (2, 7, 8).

Bacteriophages (phages) have emerged as promising biocontrol agents due to their ability to specifically target and lyse bacterial cells (9). Their rapid bactericidal effect and high specificity for bacterial hosts draw attention to the applications in various fields, including therapeutic applications (e.g., phage therapy) (10) and food safety (11, 12). Phage infection initiates by recognizing the receptor of the host bacteria through its tail fibers (13). In many tailed phages, receptor-binding proteins (RBPs) located at the distal tip of their tail proteins mediate binding to the specific receptors on the bacterial surface (13, 14). Even minor mutations in RBPs can significantly influence the recognition of specific bacterial surface receptors such as lipopolysaccharide (LPS) or outer membrane proteins and teichoic acids (15–17). These alterations may result in differences in infectivity or expanded host spectrum.

LPS, a crucial virulence factor in Gram-negative bacteria, frequently serves as the receptor for phages (13, 18–20). LPS consists of three components, including lipid A, the core region, and the O-antigen (21). The highly variable O-antigen allows pathogens to evade the host immune system (22). To date, 17 serotypes of O-antigen have been identified in *Cronobacter*, including seven serotypes specific to *C. sakazakii* (23). Since the phages that recognize the O-antigen as their receptor are typically constrained by serotype specificity (24), the high variability of the O-antigen in *C. sakazakii* strains may hinder the effectiveness of phage-based biocontrol strategies. To overcome these barriers, a comprehensive understanding of the molecular interactions between *C. sakazakii* phages and their bacterial host is important, but it remains poorly characterized.

In this study, two lytic phages infecting *C. sakazakii,* named CRES7 and CRES9, were newly isolated from sewage. Genomic analysis revealed that the two phages exhibit high genomic homology, differing by only two nucleotides in the gene encoding a putative tail fiber, which may be involved in the receptor binding. Despite these minimal variations, the phages showed dissimilar host ranges due to the differential recognition of specific O serotypes of *C. sakazakii*. Elucidating the mechanism of differential recognition of receptors by mutations in the tail fiber gene can provide a foundation for developing better phage-based interventions, such as designer phages with expanded host ranges or enhanced specificity for targeted biocontrol of *C. sakazakii*.

## RESULTS

### Isolation and morphology of CRES7 and CRES9

**Fig 1:**
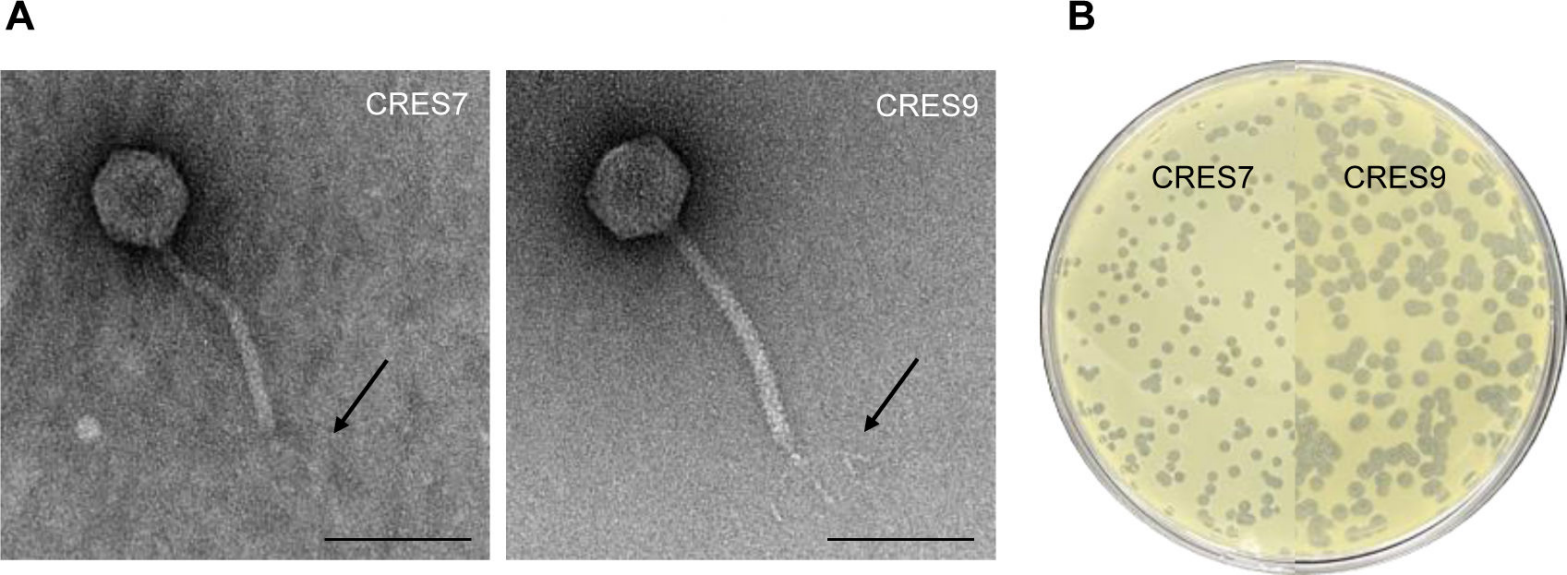
Morphological analysis of *C. sakazakii* phage CRES7 and CRES9. (**A**) TEM analysis of CRES7 and CRES9. The arrows represent the tail fiber of each phage. The scale bar indicates 100 nm. (**B**) Plaque morphology of CRES7 (left) and CRES9 (right).

Two *C. sakazakii* phages, named CRES7 and CRES9, were isolated from a sewage sample obtained from a sewage treatment plant in South Korea, using *C. sakazakii* ATCC 29544 as a host strain. Transmission electron microscopy (TEM) analysis revealed that both CRES7 and CRES9 exhibited a typical siphovirus morphotype with similar dimensions (Fig. 1A) (25). CRES7 and CRES9 had icosahedral head sizes of 78.2 ± 3.1 nm and 75.5 ± 1.6 nm, and non-contractile tail lengths of 167.9 ± 4.3 nm and 171.0 ± 3.5 nm, respectively (Fig. 1A; Table S1). CRES7 formed smaller plaques (1.7 ± 0.1 mm) compared to CRES9, which produced plaques of 2.8 ± 0.1 mm (Fig. 1B; Table S1).

### Genomes of CRES7 and CRES9 exhibit only two nucleotide variations

The genomes of CRES7 and CRES9 were composed of circular double-stranded DNA, each with a total length of 49,065 bp and a GC content of 50.04% (Fig. S1; Table S2). Of 78 predicted open reading frames (ORFs), 61 were annotated as encoding hypothetical proteins. The other ORFs were categorized into various phage functions, including phage packaging (terminase), phage structure (tape-measure protein, minor tail protein, tail assembly protein, and tail fiber), DNA replication and manipulation (exodeoxyribonuclease, recombinase, DNA primase, DNA helicase, DNA methylase, and polynucleotide kinase), host lysis (endolysin), and additional function (EaA protein) (Fig. S1). The phylogenetic analysis of the whole genome revealed that both phages belong to the *Drexlerviridae* family and are closely related to *Cronobacter* phage CS01 at the amino acid level (Fig. S2; Table S3). No genes related to tRNA, lysogenic conversion, virulence factors, or antibiotic resistance were identified.

**Fig 2:**
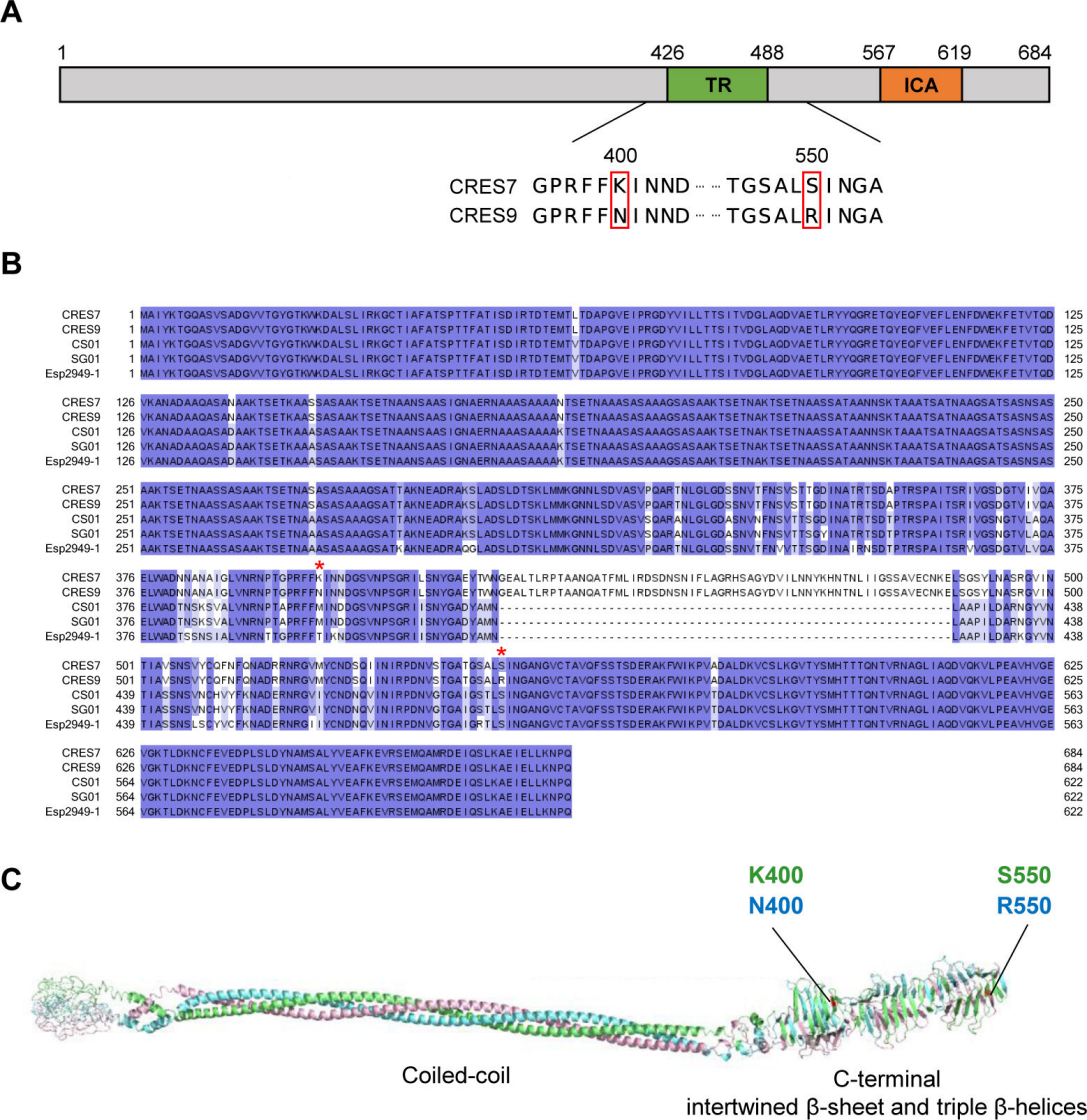
Tail fiber gp28 in CRES7 and CRES9. (**A**) Schematic domain architecture of gp28 highlighting a tail fiber protein trimerization region (TR, green, InterProScan number: IPR048388) and intramolecular chaperone auto-processing domain (ICA, orange, InterProScan number: IPR030392). The amino acid sequence alignment of gp28 in CRES7 and CRES9 is indicated below the schematic diagram. Residues 400 and 550 are highlighted in red boxes. (**B**) Sequence conservation of gp28 in related *Cronobacter* phages. Multiple sequence alignment of the gp28 tail fiber from CRES7 and CRES9, compared with gp28-like sequences from *Cronobacter* phages (YP_009814969.1 in CS01, WDS30446.1 in SG01, and YP_007005418.1 in Esp2949-1). Amino acid sequences are colored based on the percentage of identity. Residues 400 and 550 in CRES7 and CRES9 are highlighted in red asterisks. (**C**) The AlphaFold2-predicted structure of gp28 post-ICA cleavage is shown in cartoon representation, with each monomer displayed in distinct colors. The predicted gp28 structure exhibits a trimeric protein conformation featuring a coiled-coil and C-terminal intertwined β-sheet and triple β-helix. Residues 400 and 550 are highlighted in red and orange, respectively. The corresponding residues in CRES7 are illustrated in green, and those in CRES9 are shown in blue.

The two phages shared remarkably high DNA homology except for only two nucleotides. The two distinct nucleotides are located in gene *28*, which encodes a putative tail fiber (gene product 28, gp28). These variations resulted in the amino acid changes at residues 400 and 550, with K400 and S550 in CRES7 and N400 and R550 in CRES9 (Fig. 2A). BLASTp analysis revealed that gp28 has a high similarity with tail fibers of other *C. sakazakii* phages, including those of CS01 (identity, 83.48% for CRES7 and 83.33% for CRES9; accession number YP_009814969.1), SG01 (identity, 83.33% for CRES7 and 83.19% for CRES9; accession number WDS30446.1), and Esp2949-1 (identity, 82.89% for CRES7 and 82.75% for CRES9; accession number YP_007005418.1) (Fig. 2B). These results suggest that either one of these two phages might be originated from the other. Further analysis was focused on gp28.

### Gp28 in CRES7 and CRES9 is a putative tail fiber associated with receptor binding

Gp28 in CRES7 and CRES9 was predicted to function as an RBP by the PhageRBP detection tool (26) (data not shown). The conserved domain analysis revealed that gp28 contains a tail fiber protein trimerization region (InterProScan number: IPR048388), commonly identified in the tail fibers of T4-like phages, as well as an intramolecular chaperone auto-processing (ICA) domain (InterProScan number: IPR030392), which facilitates protein folding and trimerization of tail fibers before autoproteolytic cleavage (Fig. 2A) (27). These structural motifs are commonly observed in the tail fibers of various phages, including *Salmonella* phage S16 (28), coliphage T5 (29), and K1F (27).

AlphaFold2 predicted that gp28 forms a trimeric extended structure consisting of coiled-coil and C-terminal intertwined β-sheet and triple β-helices (Fig. 2C) as observed in the tail fibers of other phages (30, 31). The two distinct amino acids at residues 400 and 550 in CRES7 and CRES9 are located within the C-terminal β-helix repeats and exposed on the surface (Fig. 2C; Fig. S3), suggesting that they can potentially affect receptor binding of gp28 differentially (Fig. S3). These structural predictions suggest that CRES7 and CRES9 may show different receptor binding, which can lead to different host ranges even though the only difference between the two phages is two amino acid residues in gp28.

### Both CRES7 and CRES9 use the O-antigen as their host receptor but exhibit different host ranges

The tail module of *Drexlerviridae* phages consists of lateral and central tail fibers (32, 33). Maffei et al. (32) suggest that the lateral tail fiber likely binds to LPS, while the central tail fiber, analogous to gpJ of phage lambda, generally attaches to outer membrane proteins, including FhuA, BtuB, and TolC. To identify the receptors of CRES7 and CRES9, spotting assays were performed on *C. sakazakii* mutants lacking either one of the genes encoding known phage receptors, including LPS, flagella, and outer membrane proteins (14, 18, 34, 35). Only the *waaL* deletion mutant, which lacks the O-antigen (36), showed resistance to both CRES7 and CRES9 (Table S4). Complementation of the *waaL* mutation restored the phage susceptibility, confirming that both CRES7 and CRES9 use the O-antigen as their host receptor (Table S4). LPS alone can trigger genome ejection in phages such as P22 (37) and 9NA (38), which are known to use LPS as a receptor. To determine whether CRES7 and CRES9 require secondary receptors, an *in vitro* DNA ejection assay was performed. If LPS was sufficient to induce DNA release, the number of infectious virion particles would be reduced following incubation with LPS compared to the control without LPS. We found that LPS alone triggered DNA ejection in both CRES7 and CRES9, confirming LPS as a sole receptor for both phages (Fig. S4).

To evaluate whether specific amino acid variations in the tail fiber influence host specificity, host ranges of both phages were tested against 24 *C*. *sakazakii* strains, including 3 type strains and 21 strains isolated from food samples (39). CRES9 was able to infect 19 out of 24 strains (79%), whereas CRES7 infected only 11 strains (46%) (Table 1). Neither phage could infect bacteria from the other genera, demonstrating their specificity for *C. sakazakii* (Table S5). These results demonstrated that two amino acid differences in the tail fiber gp28 can significantly influence the host ranges of CRES7 and CRES9.

**TABLE 1:** Host range of phages CRES7 and CRES9

Bacterial strain	Phage^*a*^		Reference or source
	CRES7	CRES9	
*C. sakazakii* type strains			
ATCC 29544	+	+	ATCC^*b*^
ATCC BAA-894	I	I	
ATCC 29004	‒	‒	
*C. sakazakii* isolates (sample origin)			
4-1 (dried fish)	‒	+	(39)
4-2 (dried fish)	‒	+	
4-3 (dried fish)	‒	+	
5-2 (dried fish)	‒	+	
5-3 (dried fish)	‒	+	
5-4 (dried fish)	‒	+	
15-1 (sangsik)	+	+	
15-2 (sangsik)	+	+	
17-1 (sangsik)	‒	‒	
17-2 (sangsik)	‒	‒	
17-3 (sangsik)	+	+	
18-1 (sangsik)	+	+	
18-2 (sangsik)	+	+	
18-3 (sangsik)	+	+	
19-2 (sangsik)	+	+	
19-3 (sangsik)	+	+	
22-1 (sunsik)	‒	+	
22-2 (sunsik)	‒	+	
22-3 (sunsik)	+	+	
31-2 (sunsik)	‒	‒	
31-3 (sunsik)	+	+	

^*a*^ +, plaque formation; I, formation of inhibition zone; ‒, no infection.

^*b*^ ATCC, American Type Culture Collection.

### CRES7 and CRES9 can infect different O serotypes of *C. sakazakii*

Since both CRES7 and CRES9 use the O-antigen as a receptor and the predicted structure revealed that two different amino acid residues are located on the surface of the tail fiber, we hypothesized that the specific residues in gp28 may contribute to differential recognition of various structures of O-antigen, affecting the host range of phage. To investigate this hypothesis, we performed PCR-based O serotyping on nine representative *C. sakazakii* strains exhibiting distinct susceptibilities to CRES7 and CRES9, as shown in Table 2. The nine *C*. *sakazakii* strains could be classified into three different serotypes: four isolates, including 22-3, 31-3, 15-2, and 15-1 are serotype O1; three isolates, including 5-2, 4-1, and 22-1 are serotype O3; and one isolate 31-2 is serotype O2 (Table 2). These results showed a strong correlation between O serotype and phage infectivity. The *C. sakazakii* strains susceptible to both CRES7 and CRES9 were all serotype O1, while the strains susceptible only to CRES9 were serotype O3 (Table 2). The strain resistant to both phages was serotype O2 (Table 2).

**TABLE 2:** LPS O serotypes of *C. sakazakii* strains

*C. sakazakii* strains	Phage^*a*^		O serotype
	CRES7	CRES9	
ATCC 29544	+++	+++	O1
Isolate 22-3	+++	+++	O1
Isolate 31-3	++	++	O1
Isolate 15-2	+	+	O1
Isolate 15-1	+	+++	O1
Isolate 5-2	‒	+++	O3
Isolate 4-1	‒	++	O3
Isolate 22-1	‒	+	O3
Isolate 31-2	‒	‒	O2

^*a*^ +++, efficiency of plating (EOP) 0.1–1; ++, EOP 0.001–0.01; +, EOP <0.001; ‒, no infection.

**Fig 3:**
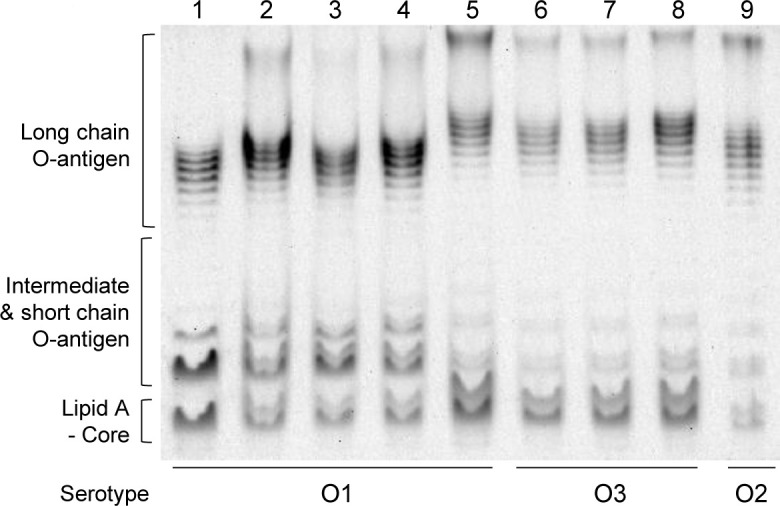
Analysis of LPS profiles of *C. sakazakii* strains. The profiles of LPS extracted from *C. sakazakii* strains. LPSs were extracted using the hot-phenol extraction method and separated on 15% polyacrylamide gel. Lane 1, *C. sakazakii* ATCC 29544; lane 2, *C. sakazakii* isolate 22-3; lane 3, isolate 31-3; lane 4, isolate 15-2; lane 5, isolate 15-1; lane 6, isolate 5-2; lane 7, isolate 4-1; lane 8, isolate 22-1; lane 9, isolate 31-2. Serotype O1, lanes 1–5; serotype O3, lanes 6–8; and serotype O2, lane 9.

Both phages showed similar efficiency of plating (EOP) on *C. sakazakii* O1 strains, except for isolate 15-1. This led us to analyze the LPS profiles by comparing the modal distribution of their O-antigen chain lengths. Three strains in serotype O1 (isolates 22-3, 31-3, and 15-2) displayed O-antigen profiles similar to those of ATCC 29544, which was used as a reference strain (Fig. 3, lanes 1–4). These strains showed comparable EOP values for both CRES7 and CRES9. However, isolate 15-1 had longer O-antigen chains than the other O1 strains (Fig. 3, lane 5), and CRES9 showed a higher EOP on isolate 15-1 than CRES7 (Table 2). Three strains in serotype O3 (isolates 5-2, 4-1, and 22-1), which were resistant to CRES7 but susceptible to CRES9, also exhibited longer O-antigen chains than O1 strains (Fig. 3, lanes 6–8). *C. sakazakii* isolate 31-2, which was resistant to both phages, had intermediate modal lengths compared to serotypes O1 and O3 (Fig. 3, lane 9). These results suggest that residues 400 and 550 of gp28 can recognize and differentiate O-antigen structures, even within the same serotype.

### CRES7 and CRES9 exhibit different adsorption rates and burst sizes

**Fig 4:**
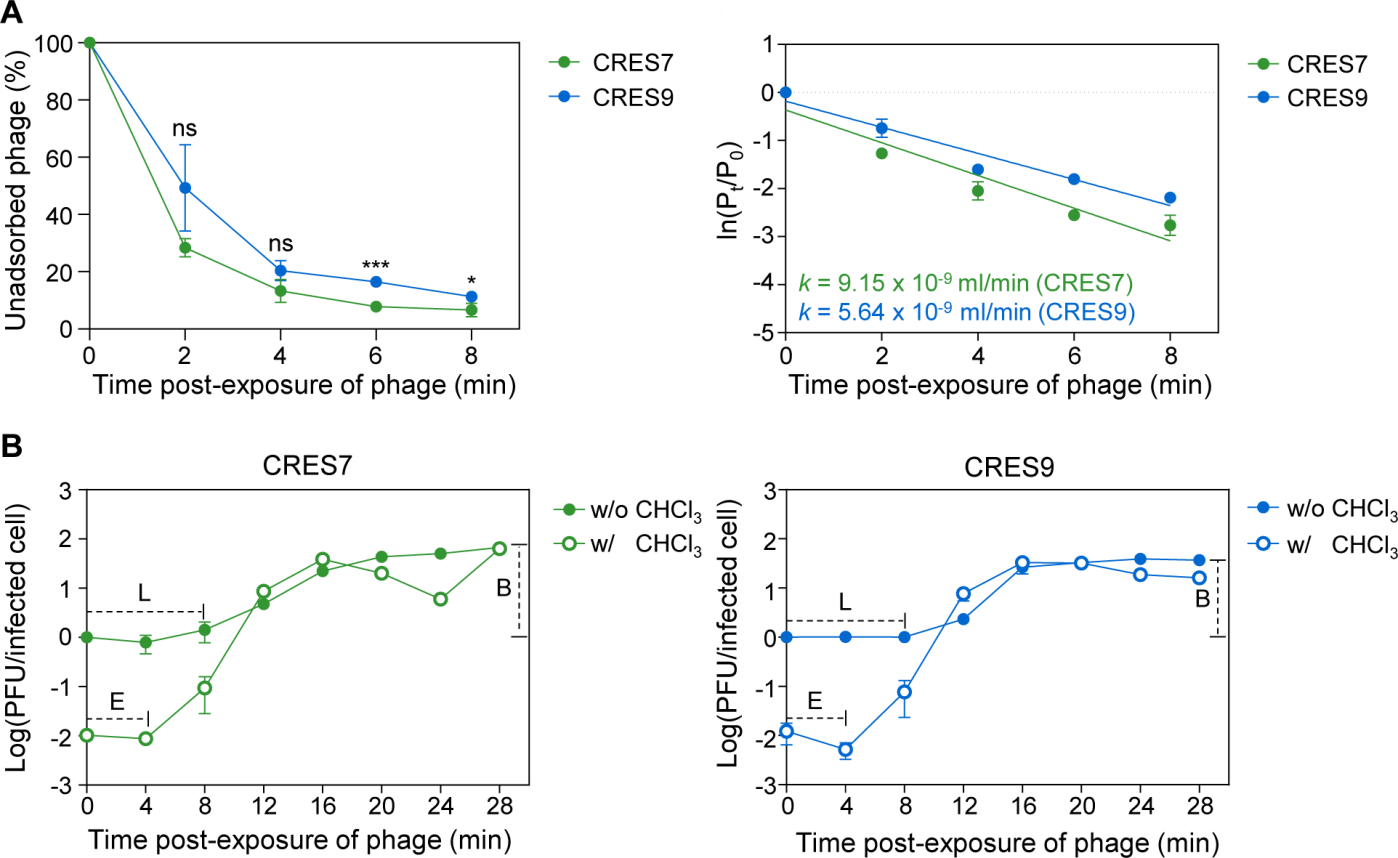
Phage infection and replicative characteristics of CRES7 and CRES9 in *C. sakazakii* ATCC 29544. (**A**) The phage adsorption rate of CRES7 (green) and CRES9 (blue) is plotted in the left panel. The adsorption kinetics are plotted in the right panel. The data represent the means with standard deviations from three independent experiments. Statistical analysis was performed by using Student’s *t*-test, using GraphPad Prism version 8.0.1 (*P < 0.05 and ***P < 0.001). (**B**) One-step growth curve of CRES7 (left) and CRES9 (right). E, eclipse period; L, latent period; B, burst size. The data represent the means with standard deviations from three independent experiments.

Given that variations in receptor recognition can influence phage adsorption efficiency, the adsorption rates of CRES7 and CRES9 were compared. On *C. sakazakii* ATCC 29544, CRES7 exhibited a higher adsorption rate than CRES9, with 93% of CRES7 adsorbing to host cells within 8 min post-infection, compared to 83% for CRES9 (Fig. 4A). Additionally, the adsorption constant (*k*) for CRES7 was 162% higher than that of CRES9, indicating greater binding efficiency to ATCC 29544 (Fig. 4A).

Adsorption efficiency also varied depending on the O serotype. Both phages exhibited reduced adsorption efficiency toward the O3 strain in the adsorption assay, with CRES9 adsorbing more efficiently than CRES7 (Fig. S5). This finding suggests that the gp28 of CRES7 may have a lower binding affinity for O3 LPS, resulting in reduced adsorption. Notably, while minimal adsorption of CRES7 to the O3 strain was observed, this did not lead to productive infection, indicating that the level of adsorption was insufficient to initiate infection (Fig. S5). Neither phage exhibited detectable adsorption within 90 min for the O2 strain, suggesting a lack of interaction with O2 LPS (Fig. S5). These findings indicate that residues 400 and 550 in gp28 influence adsorption efficiency and specificity for different O serotypes.

To assess whether the differences in adsorption influenced phage propagation, one-step growth curves were compared. Both CRES7 and CRES9 exhibited similar latent periods of approximately 8 min and eclipse periods of approximately 4 min (Fig. 4B). However, CRES7 had a notably larger burst size, producing 57.6 PFU per infected cell—approximately twice as many as CRES9 (30.3 PFUs per infected cell) (Fig. 4B). These results suggest that differences at residues 400 and 550 in tail fiber gp28 influence both adsorption and phage burst size.

### CRES7 and CRES9 exhibit similar antibacterial activity and thermal/pH stability

**Fig 5:**
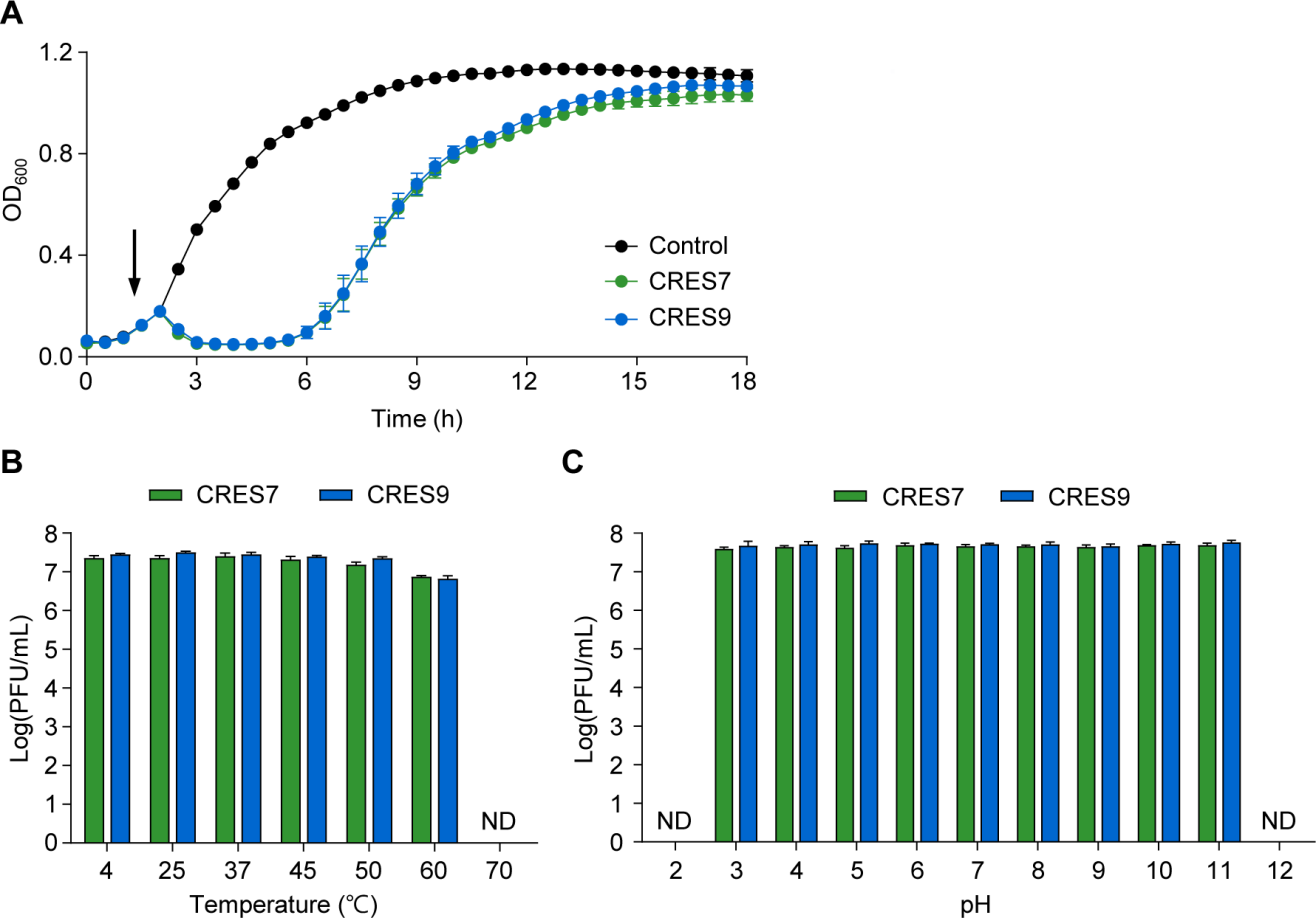
The lysis activity and the thermal and pH stability of CRES7 and CRES9 in *C. sakazakii* ATCC 29544. (**A**) The challenge assay of CRES7 (green) and CRES9 (blue) at an MOI of 0.1. The phages were infected at the time indicated with an arrow. The same volume of SM buffer was used as a negative control. (**B and C**) The thermal (**B**) and pH (**C**) stability of phages. The data represent the means with standard deviations from three independent experiments. ND, not detected.

To analyze the impact of gp28 variations on the antibacterial activity, the bacterial challenge assay was performed. Both CRES7 and CRES9 reduced bacterial growth within 1 h and continued to inhibit *C. sakazakii* ATCC 29544 growth for up to 5 h post-infection at a multiplicity of infection (MOI) of 0.1 (Fig. 5A). No significant differences in bacterial growth inhibition patterns were observed between the two phages in *C. sakazakii* ATCC 29544, indicating that the gp28 variations do not affect overall lytic activity.

Because some mutations in RBPs can affect thermodynamic stability by destabilizing the protein’s structural integrity (40, 41), the thermal and pH stability of CRES7 and CRES9 were determined. Thermal stability assays showed that both phages remained viable at temperatures up to 60°C but lost infectivity at 70°C (Fig. 5B). Similarly, both phages maintained stability across pH ranges of 3–11 but were inactivated at extreme pH levels (pH 2 and pH 12) within 1 h (Fig. 5C). These results indicate that the gp28 variations do not affect the thermal and pH stability of CRES7 and CRES9.

## DISCUSSION

Tail fibers play a crucial role in the initial recognition and binding to bacterial receptors (14). Even minor amino acid substitutions in the tail proteins can drastically affect host recognition and change the host range, as observed in experimental evolution (42) and phage engineering studies (15, 17). For example, a single point mutation in the tail fiber ORF31 of *Enterococcus* phage ΦEF24C significantly increased its adsorption and infectivity (42). In coliphage T4, point mutations in the long tail fiber altered its binding affinity to outer membrane porin C (OmpC) and LPS, changing its host range (15). Similarly, a single mutation in the tailspike protein gp14 of *Shigella* phage Sf6 expanded the host range (17).

In this study, we demonstrated that the two naturally occurring amino acid variations in the tail fiber gp28 of *C. sakazakii* phages, CRES7 and CRES9, influenced host recognition through differential binding of LPS O-antigens (Tables 1 and 2). CRES7, which carries K400 and S550 in gp28, infected only *C. sakazakii* O1 strains, whereas CRES9, which has N400 and R550 in gp28, infected both *C. sakazakii* O1 and O3 strains (Tables 1 and 2). *C. sakazakii* O1 is prevalent in powdered infant formula and is frequently associated with clinical cases of *C. sakazakii* infections (43, 44). Although *C. sakazakii* O3 is less common in clinical cases, it is increasingly isolated in food-processing environments (43, 44). This highlights the importance of phages with a broader host range, like CRES9, in enhancing phage-based interventions aimed at controlling *C. sakazakii* in food safety applications.

Amino acid residue 400 of gp28 is located near several aromatic amino acid residues (F398, F399, Y417, Y421, and W423) on the surface of the C-terminal β-helix. These aromatic residues may play crucial roles in O-antigen binding, as protein-saccharide interactions typically involve the stacking of sugar moieties onto the aromatic side chains (45). Following the maturation of tail fiber, residue 550 is located at the distal tip of the C-terminal domain (Fig. S3), which is known to interact with bacterial receptors in many phages (15, 46, 47). The substitution of polar uncharged amino acids with positively charged residues at these key positions is likely to significantly impact electrostatic interactions and binding affinity to the O-antigen, ultimately leading to the alteration of the phage host range. For instance, a point mutation at the C-terminus of tail fiber ORF84 (K653N) of *Pseudomonas* phage JG004 expanded its host range (48), while a mutation in the long tail fiber adhesin of *Cronobacter* phage Pet-CM3-1 (K163Q) reduced its host range (49).

The different susceptibilities of *C. sakazakii* isolate 15-1 to CRES7 and CRES9 further underscore the importance of the two residues in host recognition (Table 2). Although classified as serotype O1, *C. sakazakii* isolate 15-1 exhibited a distinct O-antigen structure compared to the other O1 strains (Fig. 3). While isolate 15-1 shares genetic similarity in the *wzy* gene (encoding O-antigen polymerase) with other O1 serotype strains, its LPS O-antigen may differ due to specific structural variations. In *Escherichia coli*, some serogroups with highly similar O-antigen gene clusters can synthesize distinct O-antigen structures (50). For example, O107 and O117 share 98.6% overall DNA identity in their O-antigen gene clusters and contain the same genes in the same organization, yet O107 has a substitution of D-GlcNAc for D-Glc in O117 (51). Similarly, isolate 15-1 may contain modifications, such as acetylated glycosides, on the LPS. These modifications might selectively alter the gp28 binding affinity, resulting in differences in infection efficiency between CRES7 and CRES9. These findings indicate that residues 400 and 550 contribute to recognizing subtle O-antigen variations, even among strains classified as the same O serotype.

Furthermore, adsorption kinetics on *C. sakazakii* strain ATCC 29544 varied between the two phages, with CRES7 exhibiting a significantly higher adsorption rate than CRES9 (Fig. 4). The faster adsorption of CRES7 may be associated with its formation of smaller plaques (Table S1), as the rapid binding limits diffusion before attachment, thereby limiting plaque expansion (52). These results highlight the importance of residues 400 and 550 in receptor recognition and adsorption dynamics.

CRES7 produced approximately twice as many progenies as CRES9 (Fig. 4B). The increase in burst size is directly related to the extension of lysis time (53). However, no significant differences in the latent and eclipse periods between CRES7 and CRES9 were detected, as shown in Fig. 4B. Other factors, including the efficiency of translational and phage assembly processes, may also contribute to the observed differences in burst size. The two amino acid differences may influence the stability of the gp28 trimer, potentially affecting its interaction with other structural components during virion assembly.

Both CRES7 and CRES9 were isolated from the same environmental sample, highlighting their natural coexistence. This observation underscores the significance of our findings, as these two phages have naturally adapted distinct tail fiber variations without experimental manipulation. Unlike studies in which phages were engineered (54–56) or evolved under laboratory conditions to expand or shift their host range (17, 57, 58), the coexistence of CRES7 and CRES9 in the same environment suggests that natural selective pressures can independently drive adaptation in tail fibers, shaping phage-host interactions over time. This natural coexistence has greater ecological relevance, as it reflects the dynamics of the phage population in response to varying bacterial communities (59, 60). CRES9, with its broader host range, may be better suited for environments with high bacterial diversity, while CRES7, with its rapid adsorption and higher progeny yield, could have an advantage in environments with dense, low-diversity bacterial populations (59). Over time, such pressures may favor specific tail fiber residues, allowing phages with different fitness strategies to coexist within the same ecological niche.

By investigating these naturally occurring differences in amino acid residues, we can improve our understanding of how these alterations contribute to phage fitness, which could help guide the development of phage-based biocontrol strategies. This study provides a foundation for further research into the adaptive processes that allow diverse phages to coexist and thrive in natural environments.

## MATERIALS AND METHODS

### Bacterial strains and culture conditions

*C. sakazakii* ATCC 29544 was used as the host bacterium for phage isolation and propagation in this study. All bacterial strains were incubated at 37°C in Luria-Bertani (LB) broth (BD Difco, NJ, USA) with shaking. For the culture of bacteria harboring a plasmid, ampicillin (50 µg/mL) was added to the medium.

### Phage isolation and stock preparation

Phages were isolated following a previously described protocol (61). In brief, sewage samples were collected from a sewage treatment plant in Seoul, South Korea, and filtered through 0.22 µm filters (Sartorius, Goettingen, Germany). The filtrate was enriched in LB broth containing *C. sakazakii* ATCC 29544. After centrifugation (10,000 × *g* at 4°C for 10 min) and filtration, the filtrate was streaked onto LB agar plates, and 0.4% LB soft top agar containing host bacteria was poured on it. After incubation at 37°C overnight, single plaques were collected, suspended in SM buffer (100 mM NaCl, 8 mM MgSO_4_∙7H_2_O, and 50 mM Tris-HCl, pH 7.5), and filtered. This process was repeated five times to purify a single phage isolate.

For phage propagation, the host bacterial culture in the exponential phase was infected with phage lysate and incubated at 37°C for 4 h (34). The phage lysate was collected by centrifugation and subsequent filtration. The concentrated stock solution of phage was prepared via CsCl gradient ultracentrifugation (61). The phage stocks were stored in glass vials at 4°C.

### TEM analysis

Phage morphology was analyzed by TEM as previously described (61). In brief, phage samples (10^10^ PFU) were placed onto the glow-discharged formvar/copper grids and stained using 2% uranyl acetate (pH 4.0). The images of phage samples were captured using an energy-filtering TEM (LIBRA 120, Carl Zeiss, Germany) at the National Instrumentation Center for Environmental Management (Seoul, South Korea). Head and tail dimensions were measured using the ImageJ program (*n* = 5).

### Whole-genome sequencing of phage genomic DNA

Phage genomic DNA was extracted using a phenol-chloroform extraction method as described previously (61). Whole-genome sequencing was performed using Illumina Miseq and assembled using SPAdes version 3.15.2. The ORFs and their functions were predicted using GeneMarkS (62) and RAST (63), BLASTp (64), and InterProScan databases (65), and each ORF was annotated manually based on the information. The genome map was visualized by using GeneScene version 0.99.8.0 (DNAstar, Madison, WI, USA). The proteomic phylogeny of phages was analyzed using ViPTree (https://www.genome.jp/viptree) (66) and visualized using VICTOR (https://victor.dsmz.de) (67). All 60 viral genomes of the *Drexlerviridae* family were selected to generate a whole-genome proteomic tree with CRES7 and CRES9 (Table S3). The Genome-BLAST Distance Phylogeny method (68) was applied for pairwise comparisons of the amino acid sequences under settings specific for prokaryotic viruses (67).

### *In silico* analysis of gp28

BLASTp (64) was performed to identify the function and the related proteins of gp28 of CRES7 and CRES9. The multiple sequence alignment was conducted by Clustal X2 (69) and visualized by Jalview software version 2.11.0 (70). The probability of RBP was analyzed by PhageRBPdetection version 2 (26). The conserved domains were analyzed using InterProScan (65) and the HHpred online server (71) with default parameters. The predicted homotrimeric structure of gp28 was generated using AlphaFold2 version 2.1.2 under the following parameters: model_preset = multimer and db_preset = full_dbs (72–74). Structures were visualized using PyMOL 2.5.2 (75).

### Host receptor determination

The host receptor determination of phages was conducted using *C. sakazakii* ATCC 29544 as wild-type (WT) and its mutant strains (34). Briefly, each phage suspension was spotted on the bacterial lawn prepared with WT or mutant strains. After incubation at 37°C for 6 h, the EOP was calculated. The complemented study was conducted at the same time (Table S4).

### *In vitro* DNA ejection assay

LPS was extracted from *C. sakazakii* ATCC 29544 using an LPS extraction kit (iNTRON Biotechnology, South Korea). The pellet was resuspended in distilled water and boiled for 2 min until fully dissolved. *In vitro* DNA ejection assay was performed as previously described (76). Phages (5 × 10^3^ PFU/mL) were incubated with LPS (50 µg/mL) at 37°C for 1 h. After incubation, 100 µL of the mixture was sampled, and the number of remaining virions was quantified by plaque assay, using a control without LPS for comparison.

### Host spectrum determination

In total, 24 *C*. *sakazakii* strains, including 3 *C. sakazakii* type strains, 21 strains that were isolated in South Korea (39), 6 Gram-negative strains, and 2 Gram-positive strains were used in the host spectrum determination (Table 1; Table S5). The bacterial lawn was prepared by pouring 0.4% LB soft agar with each bacterial culture onto the LB agar plate. After serial dilution, phage suspensions were spotted on the bacterial lawn and incubated at 37°C for 6 h. The EOP was calculated based on phage sensitivity compared to that of the propagation host.

### O serotype analysis

The O serotype determination of *C. sakazakii* strains was conducted by PCR assays, as described by Yan et al. (23). The sequences of five primer pairs used for O serotype analysis are described in Table S6. The PCR assay was conducted under the following protocol: 98°C for 10 s, followed by 30 cycles of 55°C for 15 s and 68°C for 1 min, with a final extension at 68°C for 5 min. *C. sakazakii* ATCC 29544 and ATCC 29004 were used as control strains for the serotypes O1 and O2, respectively.

### LPS extraction and analysis

LPS was extracted from an overnight culture of *C. sakazakii* strains using the hot phenol-water extraction method (77). Briefly, overnight cultures of *C. sakazakii* were washed with DPBS (Dulbecco’s PBS containing 0.15 mM CaCl_2_ and 0.5 mM MgCl_2_) and resuspended in water and pre-heated phenol solution. After incubation at 68°C for 15 min, the aqueous phases were collected after centrifugation (10,000 × *g* at 4°C for 5 min). LPS was precipitated using sodium acetate and 95% ethanol, and the pellet was dissolved in distilled water and stored at ‒20°C.

The extracted LPS from *C. sakazakii* strains was analyzed using deoxycholate-polyacrylamide gel electrophoresis on 15% acrylamide gels as described previously (77). The Pro-Q Emerald 300 Lipopolysaccharide Gel Stain Kit (Invitrogen, MA, USA) was used for staining according to the manufacturer’s protocols. The gel was visualized by using a GelDoc instrument (Bio-Rad, CA, USA).

### Phage adsorption assay

The phage adsorption assay was conducted as previously described (78). Briefly, the bacterial culture in the exponential phase was infected by phage at an MOI of 0.01 and further incubated at 37°C for 8 min. Samples were obtained at 2 min intervals and centrifuged (16,000 × *g*, 1 min, 4°C) following filtration. The filtrates were serially diluted in SM buffer and spotted on the bacterial lawn. The adsorption constant (*k*) was calculated using the following equation: *k* = −ln (*P_t_*/*P*_*0*_)/*Nt*: *P_t_*, phage titer at the time *t* (PFU/mL); *P_0_*, initial phage titer (PFU/mL); *N*, the number of bacterial cells (CFU/mL); and *t,* adsorption time (min) (78).

### One-step growth assay

A one-step growth assay was conducted as previously described (78). *C. sakazakii* cells in the exponential phase were infected by phages at an MOI of 0.01 for 4 min. After centrifugation, the bacterial cell was washed with fresh LB broth and incubated at 37°C with shaking. Two samples were taken at 4 min intervals for 28 min, with one of the samples being treated with 2% (wt/vol) chloroform (final concentration) to artificially liberate phage progeny. The eclipse period, which represents the time required for phage progeny production within bacterial cells, was determined by calculating the time until the sudden increase in PFUs from chloroform-treated samples. The latent period, which represents the time until the natural release of progenies through bacterial lysis, was determined in the same way as the eclipse period using untreated samples. The PFU per infected cell was calculated by dividing the number of phages at each time point by the number of the chloroform-untreated samples at 0 min (78).

### Bacterial challenge assay

*C. sakazakii* cells in the exponential phase were infected with the phage at appropriate MOIs. The infected bacterial culture was further incubated at 37°C for 24 h with shaking. The OD_600_ was measured every 30 min using a SpectraMax i3x instrument (Molecular Devices, CA, USA). SM buffer was used as a negative control.

### Thermal and pH stability test

To evaluate the thermal and pH stability, phage suspension (10^8^ PFU/mL) in SM buffer was incubated at each thermal condition (4°C, 25°C, 37°C, 45°C, 50°C, 60°C, and 70°C) for 1 h and each pH-adjusted SM buffer (pH 2, 3, 4, 5, 6, 7, 8, 9, 10, 11, and 12) for 1 h at 37°C. The number of plaques was calculated by spotting assay, and the thermal and pH stability were determined.

## Data Availability

The complete genome sequences of phages were deposited in GenBank under accession numbers ON979384 (CRES7) and ON979385 (CRES9).

## SUPPLEMENTAL MATERIAL

Supplemental material jvi.00289-25-s0001.pdf Figures S1 to S5; Tables S1 to S6.

ASM does not own the copyrights to Supplemental Material that may be linked to, or accessed through, an article. The authors have granted ASM a non-exclusive, world-wide license to publish the Supplemental Material files. Please contact the corresponding author directly for reuse.
