# Supplementary material for: Amino acid residues in the tail fiber differentiate the host specificity of Cronobacter sakazakii bacteriophage
Source: J Virol. 2025 Apr 11;99(5):e00289-25. doi: 10.1128/jvi.00289-25 (PMC12090712; doi:10.1128/jvi.00289-25)
Supplement: Supplemental material — Figures S1 to S5; Tables S1 to S6. [file jvi.00289-25-s0001.pdf]

Supplemental Figures

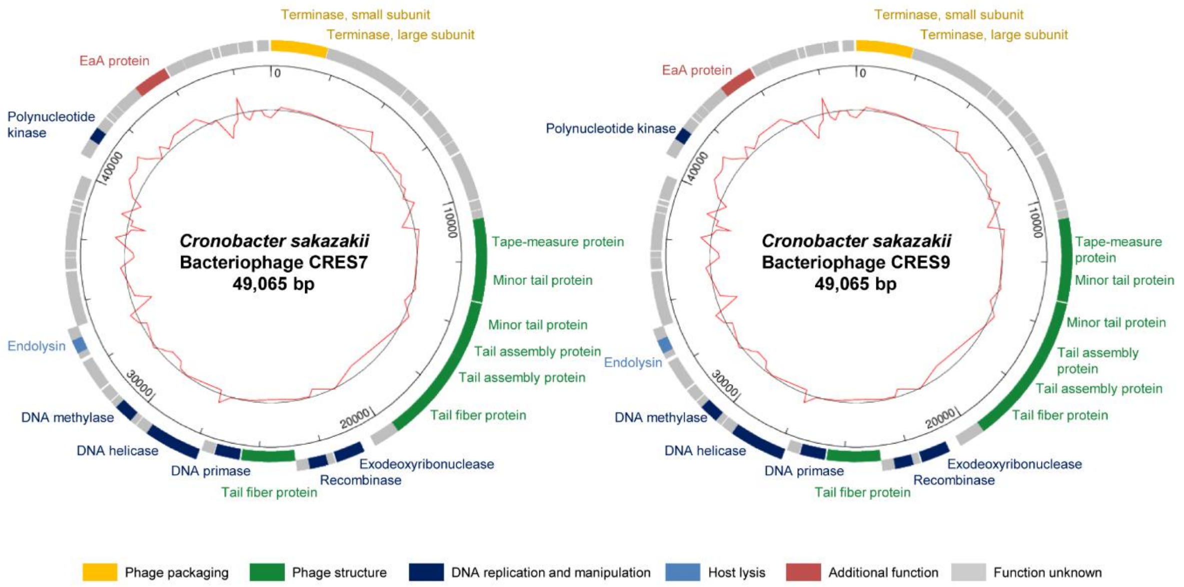

Figure S1. The genomic maps of CRES7 and CRES9. The maps were visualized using GeneScene. The colors of genes represent the functional groups: yellow, phage packaging; green, phage structure; dark blue, DNA replication, and manipulation; blue, host lysis; red, additional function; grey, function unknown.

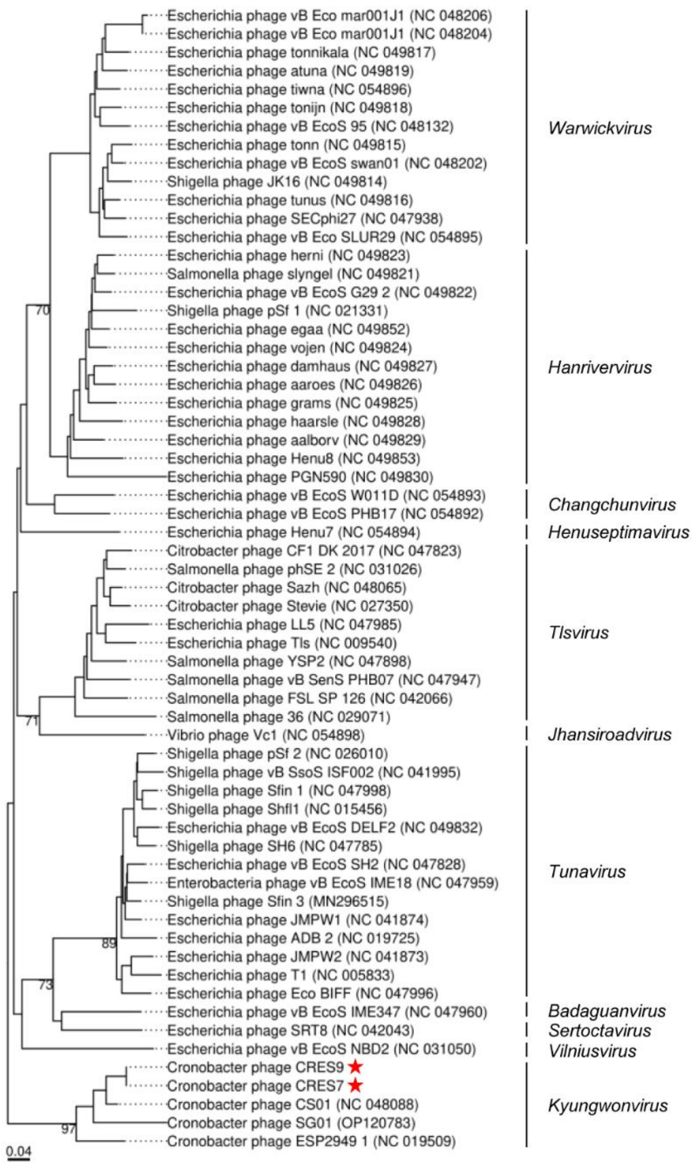

Figure S2. Phylogenetic tree of CRES7 and CRES9. The whole-genome-based proteomic trees of CRES7 and CRES9 with the 60 reference sequences of the *Drexlerviridae* family were generated using VICTOR (formula D6). Branch support was inferred from 100 pseudo-bootstrap replicates. The genus of phages is indicated.

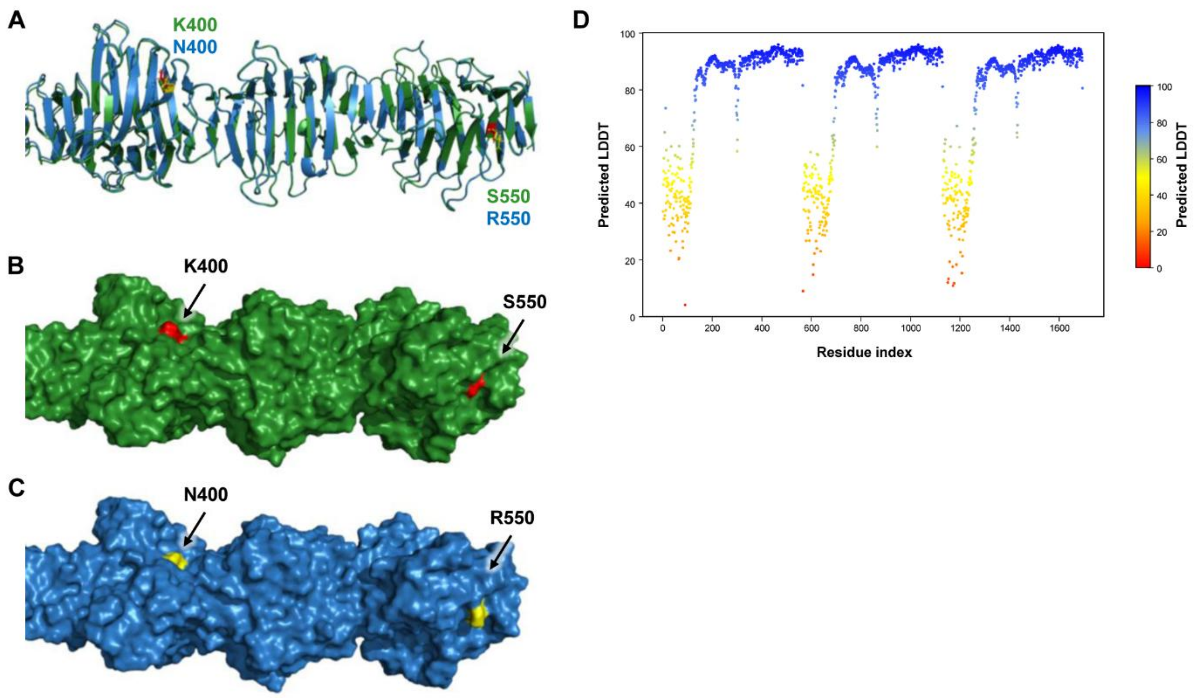

Figure S3. The location of amino acid residues 400 and 550 in gp28. (A-C) Structural predictions of the C-terminal region of gp28 from CRES7 and CRES9 generated by AlphaFold2-Multimer. (A) Structural alignment of CRES7 (green) and CRES9 (blue), with residues 400 and 550 shown in red for CRES7 and yellow for CRES9. Surface representations of CRES7 (B) and CRES9 (C). (D) The predicted Local Distance Difference Test (pLDDT) scores for the gp28 structural model shown in Fig. 2C, generated by AlphaFold2-Multimer.

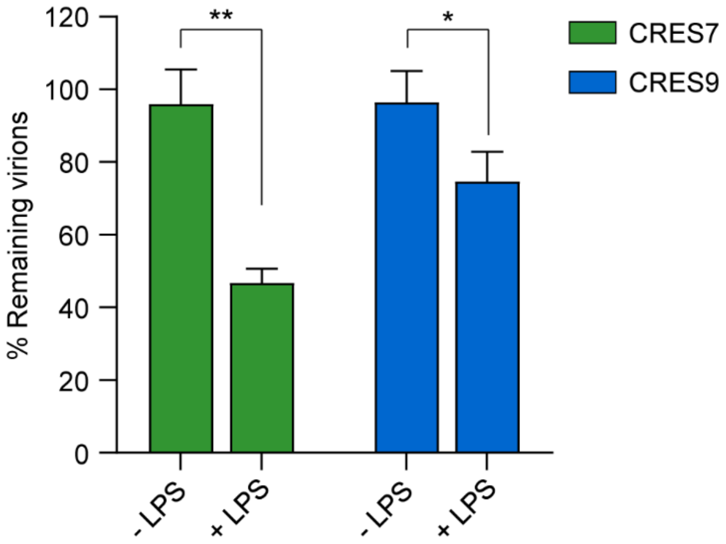

Figure S4. *In vitro* DNA ejection in CRES7 and CRES9 by LPS. Phages (5 x 10^3 PFU/ml) were incubated with LPS (50 µg/ml) extracted from *C. sakazakii* ATCC 29544 at 37°C for 1 h. The percentage of remaining virions in samples incubated without LPS (- LPS) or with LPS (+ LPS) was determined by dividing the PFU of each sample by the PFU of the initial sample. The data represent the means with standard deviations from three independent experiments. Statistical analysis was performed by using Student's *t*-test in GraphPad Prism v8.0.1 (*, $p < 0.05$; **, $p < 0.01$).

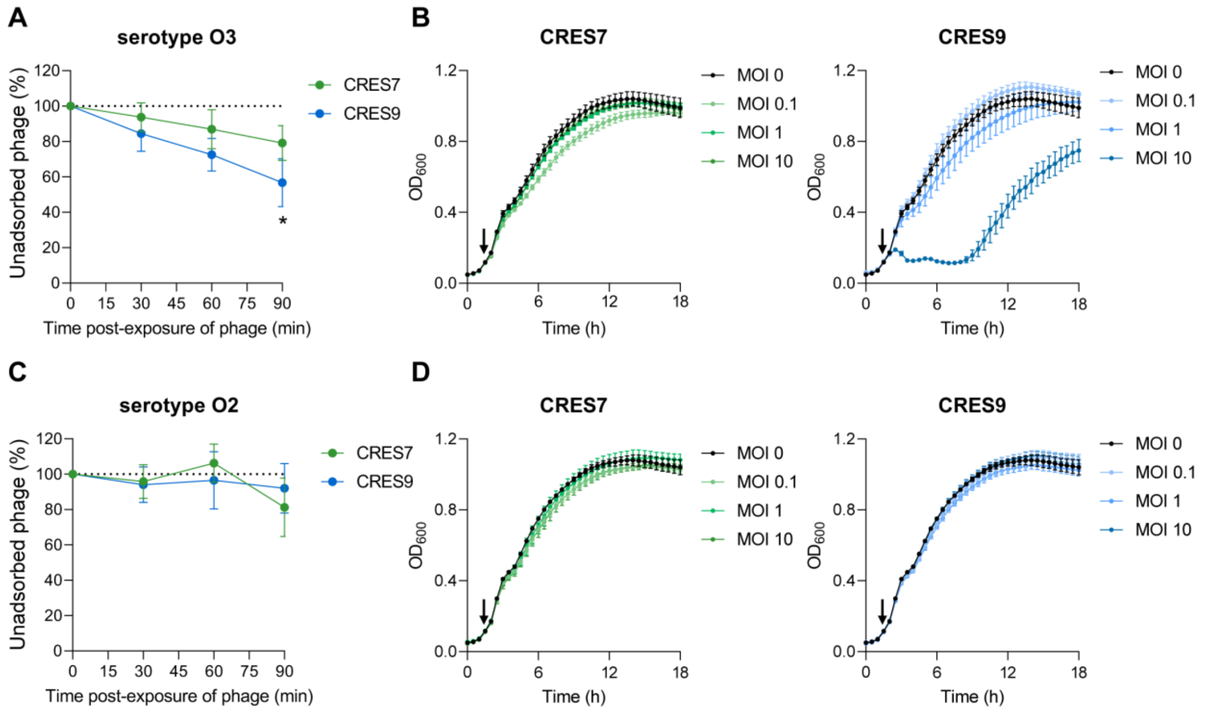

Figure S5. Phage adsorption and infectivity in *C. sakazakii* serotype O3 (A-B) and O2 (C-D). (A) The phage adsorption rate of CRES7 (green) and CRES9 (blue) in *C. sakazakii* isolate 5-2 (serotype O3) at a multiplicity of infection (MOI) of 0.001 over 90 min. (B) The challenge assay of CRES7 (green) and CRES9 (blue) in *C. sakazakii* isolate 5-2 (serotype O3) at MOIs of 0.1, 1, and 10. (C) The phage adsorption rate of CRES7 (green) and CRES9 (blue) in *C. sakazakii* isolate 31-2 (serotype O2) at an MOI of 0.001 over 90 min. (D) The challenge assay of CRES7 (green) and CRES9 (blue) in *C. sakazakii* isolate 31-2 (serotype O2) at MOIs of 0.1, 1, and 10. The phages were infected at the time points indicated by arrows. The data represent the means with standard deviations from three independent experiments. Statistical analysis was performed by using Student's *t*-test in GraphPad Prism v8.0.1 (*, $p < 0.05$).

Supplemental Tables

Table S1. Morphological analysis of phages CRES7 and CRES9

Phage	Head size (nm)	Tail length (nm)	Plaque size (mm)
CRES7	78.2 ± 3.1	167.9 ± 4.3	1.7 ± 0.1
CRES9	75.5 ± 1.6	171.0 ± 3.5	2.8 ± 0.1

Table S2. Genomic analysis of phages CRES7 and CRES9

Phage	Genomic size (bp)	GC contents (%)	ORF number	Accession number
CRES7	49,065	50.04	78	ON979384
CRES9	49,065	50.04	78	ON979385

Table S3. The NCBI Genbank accession number of complete phage genome sequences of *Drexlerviridae* used in generating phylogenetic tree

Accession number	Description
OP120783	*Cronobacter* phage SG01
NC_019509	*Cronobacter* phage ESP2949-1
NC_048088	*Cronobacter* phage CS01
NC_049823	*Escherichia* phage herni
NC_021331	*Shigella* phage pSf-1
NC_049822	*Escherichia* phage vB_EcoS_G29-2
NC_049821	*Salmonella* phage slyngel
NC_049825	*Escherichia* phage grams
NC_049827	*Escherichia* phage damhaus
NC_049852	*Escherichia* phage egaa
NC_054893	*Escherichia* phage vB_EcoS_W011D
NC_049830	*Escherichia* phage PGN590
NC_049853	*Escherichia* phage Henu8
NC_054892	*Escherichia* phage vB_EcoS_PHB17
NC_049826	*Escherichia* phage aaroes
NC_049824	*Escherichia* phage vojen
NC_048132	*Escherichia* phage vB_EcoS-95
NC_048202	*Escherichia* phage vB_EcoS_swan01
NC_049815	*Escherichia* phage tonn
NC_047938	*Escherichia* phage SECphi27
NC_049829	*Escherichia* phage aalborv
NC_049819	*Escherichia* phage atuna
NC_049818	*Escherichia* phage tonijn
NC_054896	*Escherichia* phage tiwna
NC_049817	*Escherichia* phage tonnikala
NC_049816	*Escherichia* phage tunus
NC_049814	*Shigella* phage JK16
NC_049828	*Escherichia* phage haarsle
NC_054895	*Escherichia* phage vB_Eco_SLUR29

NC_048206	*Escherichia* phage vB_Eco_mar001J1
NC_048204	*Escherichia* phage vB_Eco_mar001J1
NC_047898	*Salmonella* phage YSP2
NC_054894	*Escherichia* phage Henu7
NC_027350	*Citrobacter* phage Stevie
NC_047947	*Salmonella* phage vB_SenS_PHB07
NC_048065	*Citrobacter* phage Sazh
NC_009540	*Escherichia* phage Tls
NC_047823	*Citrobacter* phage CF1 DK-2017
NC_047985	*Escherichia* phage LL5
NC_031026	*Salmonella* phage phSE-2
NC_042043	*Escherichia* phage SRT8
NC_042066	*Salmonella* phage FSL SP-126
NC_054898	*Vibrio* virus 2019VC1
NC_047960	*Escherichia* phage vB_EcoS_IME347
NC_031050	*Escherichia* phage vB_EcoS_NBD2
NC_047828	*Escherichia* phage vB_EcoS_SH2
NC_015456	*Shigella* phage Shfl1
NC_026010	*Shigella* phage pSf-2
NC_047785	*Shigella* phage SH6
NC_041995	*Shigella* phage vB_SsoS-ISF002
NC_041873	*Escherichia* phage JMPW2
NC_047998	*Shigella* phage Sfin-1
NC_019725	*Escherichia* phage ADB-2
NC_047959	*Enterobacteria* phage vB_EcoS_IME18
NC_049832	*Escherichia* phage vB_EcoS-DELF2
NC_041874	*Escherichia* phage JMPW1
MN296515	*Shigella* phage Sfin-3
NC_047996	*Escherichia* phage Eco_BIFF
NC_005833	*Escherichia* phage T1
NC_029071	*Salmonella* phage 36

Table S4. Phage susceptibility of mutant strains for identifying host receptor

Bacterial strain and genotype	Descriptions	Phage[a]		Reference or source
		CRES7	CRES9	
***C. sakazakii* ATCC 29544**				
Wild type	Wild type	+++	+++	ATCC[b]
Δ*waaL*	O-antigen of LPS defective strain	–	–	Laboratory collection
Δ*waaL* +pwaaL[c]	O-antigen of LPS complemented in Δ*waaL* mutant strain	+++	+++	
Δ*flgK*	Flagella-defected strain	+++	+++	(1)
Δ*lamB*	Outer membrane protein LamB-defective strain	+++	+++	
Δ*ompC*	Outer membrane protein OmpC-defective strain	+++	+++	
Δ*fhuA*	Outer membrane protein FhuA-defective strain	+++	+++	
Δ*tolC*	Outer membrane protein TolC-defective strain	+++	+++	Laboratory collection
Δ*lamB*	Outer membrane protein LamB-defective strain	+++	+++	
Δ*ompX*	Outer membrane protein OmpX-defective strain	+++	+++	
Δ*ompA*	Outer membrane protein OmpA-defective strain	+++	+++	
Δ*btuB*	Outer membrane protein BtuB-defective strain	+++	+++	

[a]+++, The Efficiency of Plating (EOP) 0.1-1; –, no infection

[b]ATCC, American Type Culture Collection

[c]pwaaL, pBAD18::*waaL*

Table S5. Host range of phages CRES7 and CRES9

Bacterial strain	Phage[a]		References and sources
	CRES7	CRES9	
**Gram-negative bacteria**			
Escherichia coli K-12 substrain MG1655	–	–	ATCC[b]
Escherichia coli ATCC 43888	–	–	
Salmonella enterica serovar Typhimurium LT2	–	–	Laboratory collection
Salmonella enterica serovar Typhimurium UK-1	–	–	ATCC
Salmonella enterica serovar Enteritidis ATCC 13076	–	–	
Pseudomonas aeruginosa ATCC 27853	–	–	
**Gram-positive bacteria**			
Staphylococcus aureus ATCC 29213	–	–	ATCC
Bacillus cereus ATCC 14579	–	–	

[a]–, no infection

[b]ATCC, American Type Culture Collection

Table S6. PCR primers used for O serotype analysis

***C. sakazakii* O serotype**	**Primer name**	**Sequence (5'-3')**	**Target gene**	**Product size (bp)**	**Reference**
O1	wzyF-O1	CCCGCTTGTATGGATGTT	*wzy*	364	(2)
	wzyR-O1	CTTTGGGAGCGTTAGGTT			
O2	EsLPS2F	TCCTGCATTTGTGGATTTTGC	*wehI*	329	(3)
	EsLPS2R	AACGCATTGCGCTTGAGAAA			
O3	wzyF-O3	CTCTGTTACTCTCCATAGTGTTC	*wzy*	704	(2)
	wzyR-O3	GATTAGACCACCATAGCCA			
O4	wzyF-O4	ACTATGGTTTGGCTATACTCCT	*wzy*	890	
	wzyR-O4	ATTCATATCCTGCGTGGC			
O7	wzyF-O7	CATTTCCAGATTATTACCTTTC	*wzy*	615	
	wzyR-O7	ACACTGGCGATTCTACCC			

Reference

1. Lee J-H, Bai J, Shin H, Kim Y, Park B, Heu S, Ryu S. 2016. A novel bacteriophage targeting *Cronobacter sakazakii* is a potential biocontrol agent in foods. Applied and environmental microbiology 82:192-201.
2. Sun Y, Wang M, Wang Q, Cao B, He X, Li K, Feng L, Wang L. 2012. Genetic analysis of the *Cronobacter sakazakii* O4 to O7 O-antigen gene clusters and development of a PCR assay for identification of all *C. sakazakii* O serotypes. Applied and environmental microbiology 78:3966-3974.
3. Jarvis K, Grim C, Franco A, Gopinath G, Sathyamoorthy V, Hu L, Sadowski J, Lee C, Tall B. 2011. Molecular characterization of *Cronobacter* lipopolysaccharide O-antigen gene clusters and development of serotype-specific PCR assays. Applied and Environmental Microbiology 77:4017-4026.
